# Polyphenol-Enriched Pectin from Pomegranate Peel: Multi-Objective Optimization of the Eco-Friendly Extraction Process

**DOI:** 10.3390/molecules28227656

**Published:** 2023-11-18

**Authors:** Celina Podetti, Mathias Riveros-Gomez, María Celia Román, Daniela Zalazar-García, María Paula Fabani, Germán Mazza, Rosa Rodríguez

**Affiliations:** 1 Instituto de Ingeniería Química, Grupo Vinculado al PROBIEN (CONICET-UNCo), Facultad de Ingeniería, Universidad Nacional de San Juan, Av. Libertador San Martín (Oeste) 1109, San Juan 5400, Argentina; cpodetti@unsj.edu.ar (C.P.); mriveros@unsj.edu.ar (M.R.-G.); mcroman@unsj.edu.ar (M.C.R.); dzalazar@unsj.edu.ar (D.Z.-G.); paufabani@unsj.edu.ar (M.P.F.); rrodri@unsj.edu.ar (R.R.); 2 Instituto de Biotecnología, Facultad de Ingeniería, Universidad Nacional de San Juan, Av. Libertador San Martín (Oeste) 1109, San Juan 5400, Argentina; 3 Instituto de Investigación y Desarrollo en Ingeniería de Procesos, Biotecnología y Energías Alternativas, PROBIEN (Consejo Nacional de Investigaciones Científicas y Técnicas—CONICET and Universidad Nacional del Comahue) Buenos Aires 1400, Neuquén 8300, Argentina

**Keywords:** pomegranate peel, pectin, polyphenols, optimization

## Abstract

A multi-objective optimization was performed using response surface methodology to obtain a high-value-added product, pectin enriched in polyphenols, from pomegranate peel. For this purpose, a green extraction technique that combines citric acid and ultrasound was carried out considering three variables: time, pH, and temperature. The extraction procedure was optimized using the Box–Behnken design, these being the most suitable conditions, with an extraction time of 34.16 min, a pH of 2.2, and a temperature of 89.87 °C. At this point, the pectin yield was 31.89%, with a total retained polyphenol content of 15.84 mg GAE/g pectin. In addition, the water activity, ash content, equivalent weight, methoxyl content, and degree of esterification were determined for the pectin obtained at the optimal point. This study demonstrates that polyphenol-enriched pectin can be obtained from pomegranate peel via an eco-friendly and efficient method, and that it presents similar properties to commercial pectin, preserving its quality and with potential use as an ingredient or food supplement with a high nutritional value. This work contributes to developing sustainable strategies to valorize pomegranate agro-industrial waste and produce high-value functional ingredients.

## 1. Introduction

The world pomegranate production increased significantly from 2008 to 2017 and was estimated at 3.8 million tons in 2017 [1]. The main pomegranate-producing countries in the world are India (42.75%) and Iran (34.20%), while Spain is the main exporter of fresh pomegranate, followed by the Middle East and India and counter-seasonally by Peru and Chile in the Southern hemisphere [2]. Argentina is positioned as one of the main pomegranate producers: 4000 and 5000 tons in 2020, grown mainly in Salta, Córdoba, and San Juan [3]. During the production of pomegranate juice concentrate, the peel, seeds, and carpel membranes are separated and considered a bio-waste that represents approximately 50% of the weight of the fresh fruit [4], generating approximately 1.9 million tons of residues worldwide, whose final disposal is generally in controlled landfills. However, these bio-wastes contain pectin [5], polyphenols [6], minerals, and vitamins [7], among other compounds.

The concept of a circular economy arises as an alternative for the use of bio-waste, which aims to increase the life cycle of all materials and waste and promote recycling by treating them as resources [8,9]. Achieving widespread acceptance of a circular economy requires including everyone in society’s production and consumption patterns in a socio-technological environment [10]. Its application has been recognized as a great commercial opportunity and could promote the development of new industries and jobs, decreasing greenhouse gas emissions and increasing the efficient use of resources [8]. Considering this concept, pomegranate peel, seeds, and carpel membranes could be a raw material for the extraction of compounds with a high added value due to their industrial interest and health-beneficial properties, such as pectin and polyphenols [11]. Arun et al. [12] and Gaharwar et al. [13] summarized the possible uses of pomegranate peel for the application of the biorefinery concept, highlighting the following: biofertilizers, enzymes, bioethanol, bioactive compounds (phenolics, flavonoids, pectin, and peptides), and animal feed production. Pectin is a natural substance that is formed mainly in the primary wall and mesenchymal and parenchymal tissues of fruits and vegetables and has the function of intercellular cement. Pectin forms colloids due to its unique property of absorbing a significant quantity of water and subsequently establishing a three-dimensional structure through interactions between its acidic groups and water molecules. This, in turn, leads to the creation of a dispersed phase within a dispersing medium. The size of the dispersed particles in a colloid typically ranges from 1 to 500 nanometers, satisfying one of the crucial criteria for colloidal system formation. Furthermore, pectin belongs to the family of oligosaccharides and polysaccharides characterized by their high molecular weight, containing elongated chains composed of 1,4-α-D-galacturonic acid units. Pectin is a biopolymer that is widely used in the food industry (jams and jellies, emulsifying agent, bakery products, prebiotic properties, and stabilizing acidified milk products), packaging industry (food packaging and food coating film), and health and pharmaceutical industry (reduction in LDL cholesterol in plasma, antioxidant activity, metal-binding properties, glycemic control, encapsulating agent, and therapeutic and pharmaceutical uses) [14]. Abid et al. [5] have studied the physicochemical characteristics and gelling properties of pectin extracted from different varieties of pomegranate, and pectin yields were between 6.8% and 10.1% using a traditional method at 86 °C, 80 min, and 20 mM nitric acid. Yang et al. [15] achieved a pomegranate pectin yield of 8.5% using hot water at 86 °C and pH 1.7 for 80 min. Fırat et al. [16] found 11.56% for pomegranate pectin yield using ultrasound-assisted extraction at pH 1.5, 69 °C, and 29 min using HCl. Furthermore, dietary consumption of pectin has been shown to exhibit favorable effects in the prevention of chronic diseases such as diabetes and heart disease and to exert potential anticancer properties, which is supported by evidence from cellular and animal studies, human clinical trials, and toxicity evaluations [17,18]. 

Polyphenols are secondary metabolites of plants whose main functions in plants are to give color to flowers and fruits (mainly by anthocyanins), to give astringency to fruits and vegetables, to protect plant tissues to some extent against herbivores, and to limit the spread of pathogens in plants due to their antimicrobial activity, among other functions [19]. On the other hand, polyphenolic compounds prevent free radicals damage and have anticancer, cardioprotective, antidiabetic, antiaging, and anti-inflammatory properties [20]. The polyphenolic compounds attributed with e synergistic activities are highly effective against oxidation, peptic ulcers, myocardial infarction, tumors, and a variety of other conditions. This synergistic treatment approach holds the potential for effectively addressing a wide range of diseases [21]. Wang et al. [22] have used pomegranate peel as a dry powder for polyphenol extraction and butyric acid production. Zivkovic et al. [23] optimized the polyphenolic compounds extractions using ultrasound and obtained 80–180 mg GAE/g dw at the following conditions: time 10–60 min, ethanol concentration 10–90%, solid-to-solvent ratio 1:10–1:50, and temperature 20–80 °C. Niveditha et al. [24] investigated the effect of ultrasound pre-treatment on the total phenol content of pomegranate peel; the values were in the range of 160–225 mg GAE/100 g dw, and they recommended an ultrasound pre-treatment of 10 min and drying at 70 °C to reduce the degradation of phenolic compounds and antioxidants.

Ultrasound-assisted extraction is widely recognized as one of the most efficient extraction techniques that enhance the diffusion of solvents through cells, enabling the extraction of cellular contents once the cell walls have been ruptured. In addition to its efficiency, this technique offers significant advantages in terms of time and energy consumption compared with conventional methods. Also, the use of organic acids in conjunction with ultrasound technology constitutes an even cleaner and more environmentally friendly method [25,26,27]. Moreover, multi-objective optimization is a very useful tool in biorefinery process problems, since it allows for obtaining the best operating conditions of the process using mathematical functions. The problem consists of performing a multi-objective optimization, determining the values of a vector of variables that satisfy a set of constraints, which constitute an acceptable solution for decision making [28]. 

**Figure 1:**
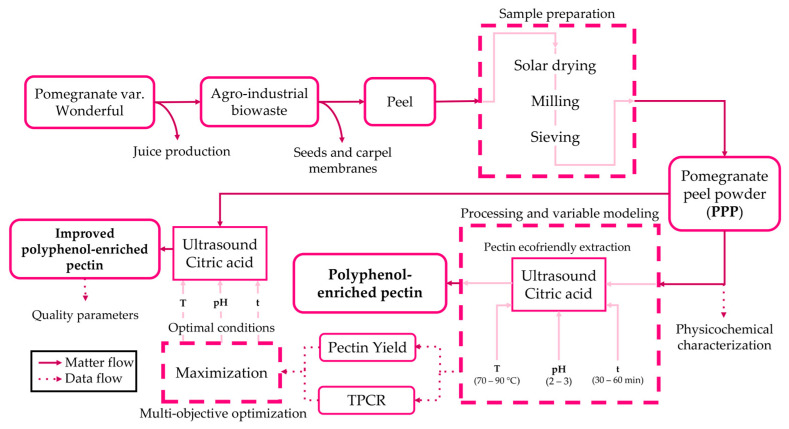
Logic diagram.

Considering the above, the objective of this work was to perform a multi-objective optimization of the extraction of pectin from pomegranate peel, simultaneously considering the pectin yield and the total content polyphenol retained in pectin, to obtain a product enriched with bioactive components by applying an alternative and eco-friendly method. It is important to remark that several works on the extraction of pectin and polyphenolic compound extractions from similar bio-wastes have been found in the bibliography [29,30,31,32]; however, no works on obtaining polyphenol-enriched pectin from any waste have been found, and this is the main novelty of this research. The extraction of the enriched pectin was carried out following an environmentally friendly process (using the citric acid solution as the solvent and ultrasound-assisted extraction). The response surface methodology (RSM) was used, applying a Box–Behnken design (BBD) to obtain a mathematical model describing the process, considering as independent variables the pH of the hydrolytic solution, the extraction temperature, and the extraction time. Figure 1 shows the scheme followed in this work.

The peel fraction derived from agro-industrial waste from juice production underwent a process involving solar drying, milling, and sieving, resulting in pomegranate peel powder (PPP). An eco-friendly pectin extraction method was employed, utilizing temperatures within the range of 70–90 °C, a pH of 2–3, and an extraction time of 30–60 min. RSM with a BBD was applied to obtain performance functions, specifically the pectin yield and the total phenolic content retained in the pectin (TPCR), as well as pectin enriched with polyphenols at different yields and concentrations. Subsequently, a multi-objective optimization approach was used to determine the optimal conditions based on the obtained pectin yield and TPCR functions. These optimal conditions were employed in the extraction process of PPP, resulting in improved polyphenol-enriched pectin, followed by the determination of its quality parameters.

## 2. Results and Discussion

### 2.1. Bio-Waste Characterization

The physicochemical characterization of the fresh peel is shown in Table 1. The pomegranate peel moisture content (60.62 ± 1.07%) was lower than the value reported by Andrade et al. (2019) for Helow var. (77.93%) [33]. However, the Sishe-Kape-Ferdos var. showed a moisture content of 46.62 ± 0.08% according to Sharayei et al. [34]. The pH and acidity are determining variables in the extraction yield of pectins, since a low pH and high acidic values favor extraction [11]. The pH (4.19 ± 0.03) and titratable acidity values (3.28 ± 0.12%) found in the analyzed samples would favor the extraction process of the pectin present in the peels. The value of soluble solids (20.00 ± 0.01 °Brix) was higher compared with the 7.6 °Brix reported by Al-Rawahi et al. [35]; the difference may be due to the variety and/or the maturity of the fruit.

Table 2 presents the physicochemical composition of pomegranate peel on a dry basis after solar drying. The moisture (6.67 ± 1.53%) was slightly higher than that reported by Arun et al. [12] (5.55%), who also used solar drying, which may be due to a shorter drying time or different environmental conditions such as air humidity and temperature that affect the equilibrium moisture content. Solar drying serves not only as a method for preserving pomegranate by-products, thereby extending their shelf life, but also for maintaining the physicochemical properties of bioactive compounds, which hold significant commercial and industrial importance [36]. The pH value was lower compared with the 4.83 reported by El Barnossi et al. [31] and higher than the 3.49 reported by Loukhmas et al. [32] for a Moroccan variety, but it was comparable to the result presented by Amir et al. [33] for pomegranates from Israel. Similarly, the measured acidity level was 6.25 ± 0.44%, which is similar to the value of 7.55 ± 0.16% reported by Urganci and Isik [37] for a Turkish variety. The ash content value was 2.69 ± 0.49%, which is lower when compared with 4.12 ± 0.10%, as reported by Urganci and Isik [34], and the 4.85 ± 0.01% reported by Muhammad et al. [38]. Regarding the aw value, it is worth noting that PPP is stable; microbial growth does not occur when a_w_ is below 0.60 [37]. The protein content closely resembled the 2.17% reported by Akuru et al. [38] for the Wonderful variety. The crude fiber content (8.16 ± 0.22%) was approximately 10% lower than the value reported by Muhammad et al. (2023) for the Kandahari variety [39]. The lignocellulosic matter content aligned with the data provided by Chaudhary et al. [40] for a Pakistani variety, with values of 28.20 ± 1.06% for hemicellulose and 36.36 ± 0.20% for the combined cellulose and Klason lignin.

### 2.2. Pectin Characterization

The pectin characteristics are tabulated in Table 3. Pectin had an a_w_ equal to 0.51 ± 0.02, similar to that obtained by Juarez-Enriquez et al. [41] in commercial pectin (0.413 ± 0.001), which ensures adequate preservation from microbial attack, increasing its shelf life. 

The ash content is related to quality and purity, since an ash content above 10% may indicate the presence of impurities or contaminants in the sample [42,43]. Therefore, the ash content (2.55 ± 0.11%) found in the enriched pectin indicates that the extracted pectin is pure. 

The EW of pectin was 175.25 ± 3.95 g/mol, which is in contrast to the values reported by other authors for orange and pomegranate peel, whose values were 614.7 and 523.2 g/mol, respectively [44]. This difference could be due to the EW, being dependent on the method of pectin extraction (pH, extracting agent, time, among others) and the nature of the raw materials [45]. The EW value is an important parameter, since it is related to the ability of pectin to form gels and indicates the amount of free, non-esterified galacturonic acid in its molecular chain. In this study, the low value may be due to the degradation of linear pectin molecules associated with the use of ultrasound during the extraction process of pectin from the raw material, leading to the formation of a weaker network [46]. Some authors obtained similar results to those of the present work, where the pectin’s EW obtained by ultrasound (343.72 ± 15.39) was lower than commercial pectin (445.59 ± 16.49) [47].

The %Me in pectin is an important factor in controlling the setting time of pectin and its ability to form gels. The % Me of pectin ranges from 0.2 to 12% depending on the source and extraction technique [14]. The value obtained in the present study (6.79 ± 0.08%) was slightly higher than the 6.15% obtained by Kareem and Naji [47]. Other authors obtained 7.1% and 9.72% for lime and orange peel, respectively [43,45]. Since the value achieved was less than 7%, the dried pectin has a low ester characteristic, implying that it is good in terms of quality. Pectin with a low methoxyl concentration creates a thermo-reversible gel, which means that it remains gelled even when heated to temperatures that would normally dissolve it. 

Finally, the DE was 41.60 ± 0.49%, classifying pomegranate-enriched pectin as low methoxyl pectin (LMP) [48,49]. The DEs from orange and pomegranate peels were 74.07 and 59.03%, respectively [47]. DE is a key molecular marker for pectin categorization, as it reflects the extent to which the carboxyl groups of pectin molecules exist as methyl esters. This is attributed to the fact that de-esterification of the polygalacturonic acid chain is favored by harsher extraction conditions [50].

### 2.3. Fitting Model and Optimization

#### 2.3.1. Development of Second-Order Polynomial Mathematical Models

Equations (1) and (2) indicate the predicted regression models for η and TPCR, respectively. These equations specify the effects of the ultrasonic extraction parameters (time, temperature, and pH) on the response variables:

Pectin yield [%] = 440.000 − 92.900 pH + 8.171 T + 0.051 T^2^ − 0.346 pH t + 0.151 pH T − 0.002 t T (1)

TPCR [mg GAE/ g pectin] = 1074.000 − 628.000 pH + 21.300 pH^2^ + 1.970 pH t + 5.491 pH T + 0.123 t T (2)

In Appendix A, a section can be observed that presents the experimental and adjusted values at each experimental condition (Table A1) and the corresponding ANOVA for the multi-variable regression performed using Fisher’s test (Table A2). The *F* values calculated, 32.18 and 13.42 for η and TPCR, respectively, were higher than the tabulated value (*F* (9,29, 0.05) = 2.22), validating the model’s adjustment. On the other hand, the coefficients of determination (R^2^) were 0.909 and 0.806 for pectin yield and TPCR, respectively.

**Figure A1:**
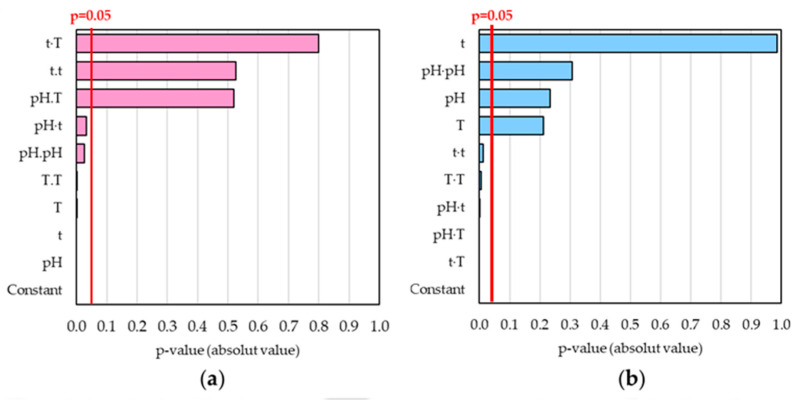
Pareto chart with p values of the statical contribution of the model terms for (**a**) η; (**b**) TPCR.

In Appendix A, the Pareto chart (Figure A1) shows the p-values for each model term, and Table A3 shows all the statistical parameter values considered here. The complete equations with non-significant terms can also be found in Appendix A.

**Figure 2:**
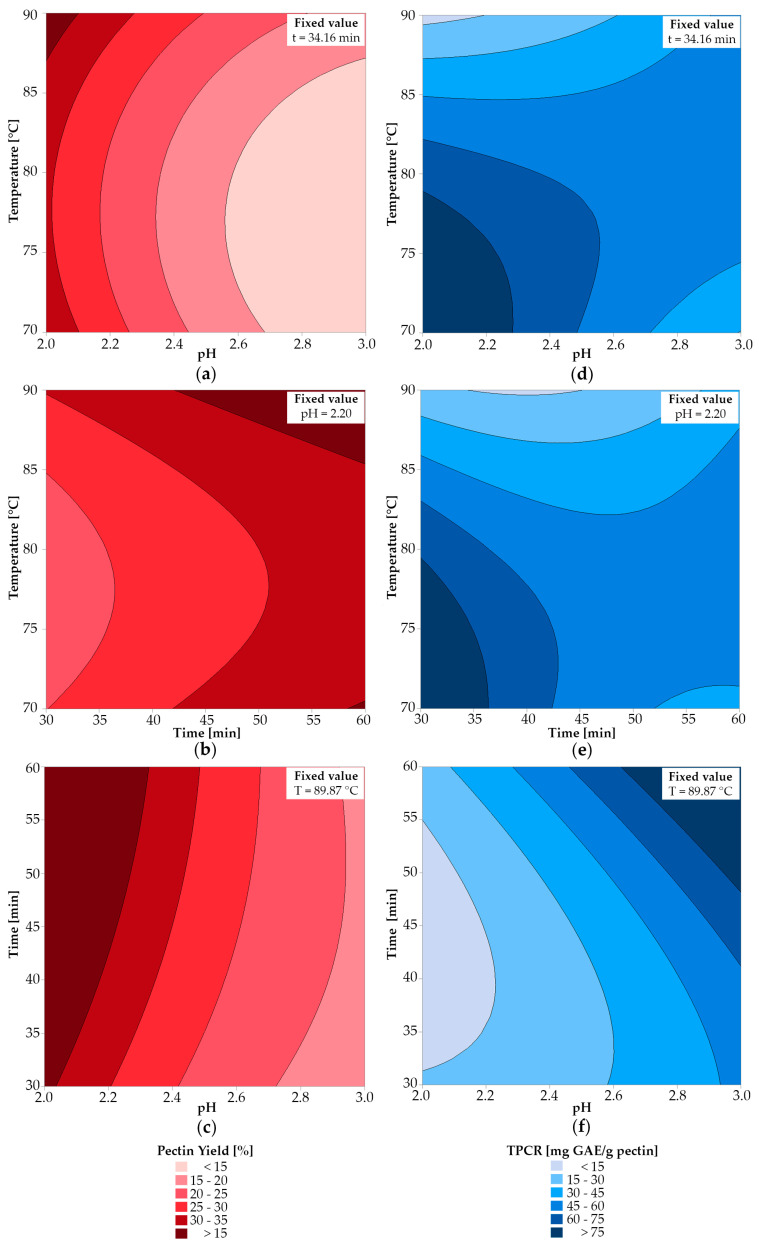
Pectin yield vs. (**a**) temperature and pH; (**b**) temperature and time; (**c**) time and pH. TPCR vs. (**d**) temperature and pH; (**e**) temperature and time; (**f**) time and pH.

Figure 2 shows the contour graphs corresponding to the adjusted mathematical models for the pectin yield (left column) and the total phenolic content retained in pectin (right column), fixing the independent variables at the optimal values indicated below in Section 2.3.2.

#### 2.3.2. Multi-Objective Optimization

**Figure 3:**
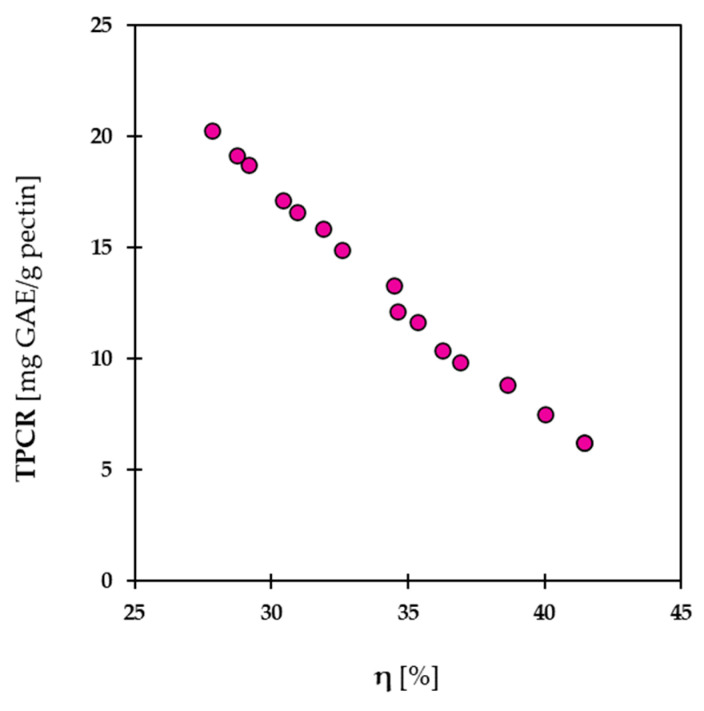
Pareto front obtained by the multi-objective optimization considering the pectin extraction yield and TPCR.

The results display a Pareto front that exhibits the optimal values, each with a balance between η and TPCR functions (Figure 3). The optimization process involved the analysis of different extraction parameters, including temperature, pH, and time, to determine the optimal conditions that maximize the process yield and the phenolic compounds retained in pectin. The independent variables at the optimal point selected take the following values: time = 34.16 min, temperature = 89.87 °C, pH = 2.20. This obtains a η = 31.89% and TPCR = 15.84 mg GAE/g pectin, achieving an excellent pectin yield while retaining a notable number of polyphenols. By considering the selected point on the Pareto front, the focus was placed on maximizing the pectin yield, which aligns with the primary objective of this study. This finding highlights the potential for maximizing the utilization of pomegranate residues, thereby reducing waste and promoting a sustainable approach in the agro-industrial sector.

#### 2.3.3. Effects of Simultaneous Variables on η and TPCR

Analyzing the contour plots (Figure 2), it is possible to see that the η ranged from 15% to 35%, while the TPCR content ranged from 15 to 75 mg GAE/g pectin. It was observed that η was favored by adverse conditions (i.e., higher temperatures, longer extraction times, and lower pH values). In addition, pH was found to have a greater effect on yield than temperature. The acid breaks down the insoluble parts of pectin, such as protopectin, and dissolves it, which increases the extraction yield. However, this increase in η occurs up to a critical pH, and at lower pH, a decrease in η is observed, which is due to the decomposition of pectin. In this study, no decrease in yield was observed, because a pH below 1.5 was not used, but Abid et al. [11] reported that the critical pH for pomegranate pectin was 1.5, at which the efficiency started to decrease. 

Concerning TPCR, it is evident that when pH is close to 2, a lower time and temperature are favorable for phenolic retention. Selahvarzi et al. [50] obtained similar results, concluding that the optimum conditions are a time between 10 and 40 min and a temperature in the range of 40–70 °C. However, Figure 2b shows that at pH 3, the retention of polyphenols is higher as the extraction time increases. One possible reason is that in pomegranate peel, the most frequently detected bioactive compounds are anthocyanins and hydrolyzable tannins, namely ellagitannins, gallotannins, flavonoids, lignans, triterpenoids, phytosterols, fatty acids, organic acids, and phenolic acids, which are very sensitive to pH changes, so at pH 3, they may be released from the matrix and be retained in the pectin in greater quantity [12,33,51,52].

Regarding the ultrasound-assisted extraction method, frequencies or sound waves between 20 kHz and 100 kHz cause pressure fluctuations in the solvent to form microbubbles that act as microjets. This facilitates the rupture of cell walls, thus improving solvent penetration and mass transfer kinetics. Therefore, this method involves less solvent and energy requirements, resulting in higher yields in shorter extraction times. These properties make this method an environmentally friendly alternative to traditional extraction methods. This method has been used to recover pectin from various fruit wastes and by-products [53]. Several reports show that this method improves the extraction yield: ultrasound extraction was applied to mango peel at 85 °C for 10 min obtaining a pectin yield of 8.1% compared with 5.4% reached with conventional heating at 85 °C for 30 min [54]. 

Some authors have attempted to establish a biorefinery process to realize a comprehensive utilization of pomegranate peel residue in order to obtain pectin and polyphenols separately, but using an enzymatic pathway [22,55]. Others have focused their studies on refining ultrasound-assisted polyphenol extraction techniques to obtain an extract with antioxidant properties [56]. Arun et al. [12] made a complete review of pomegranate peel’s multiple possible uses and remarked on the need to establish an integrated biorefinery approach. Sabater et al. [57] suggest that an integral use of pectin-rich by-products in a circular economy context should start with the recovery of compounds that are soluble in organic solvents (polyphenols, carotenoids, essential oils, etc.), followed by the recovery of pectin and subsequent use of the remaining solid (biogas, bioethanol, solid biofuel, animal feed, etc.). It is important to highlight that no reports of polyphenols being retained in pectin were found. It would be of great interest to continue this research, making an integral use where all the outflows of the extraction process are taken into account, to try to achieve the emission of zero waste.

### 2.4. Identification and Quantification of Phenolic Compounds

A total of 14 compounds were analyzed in the initial liquid extract, and in the liquid phase remaining after precipitation and filtration of the pectin, at optimal conditions. The results allowed us to determine the difference between both concentrations. The caffeic acid, catechin, chlorogenic acid, epicatechin, kaempferol, kaempferol-3-O-rutinoside, naringenin chalcone, P—coumaric acid, quercetin 3-O-glucoside, and rutin were not detected. Four phenolic compounds belonging to different classes were identified and quantitated: ellagic acid (230.27 ± 21.23 µg mL^−1^), gallic acid (15.31 ± 2.25 µg mL^−1^), punicalagin A (6.92 ± 1.48 µg mL^−1^), and punicalagin B (7.78 ± 2.09 µg mL^−1^).

## 3. Materials and Methods

### 3.1. Raw Material and Its Pretreatment

**Figure 4:**
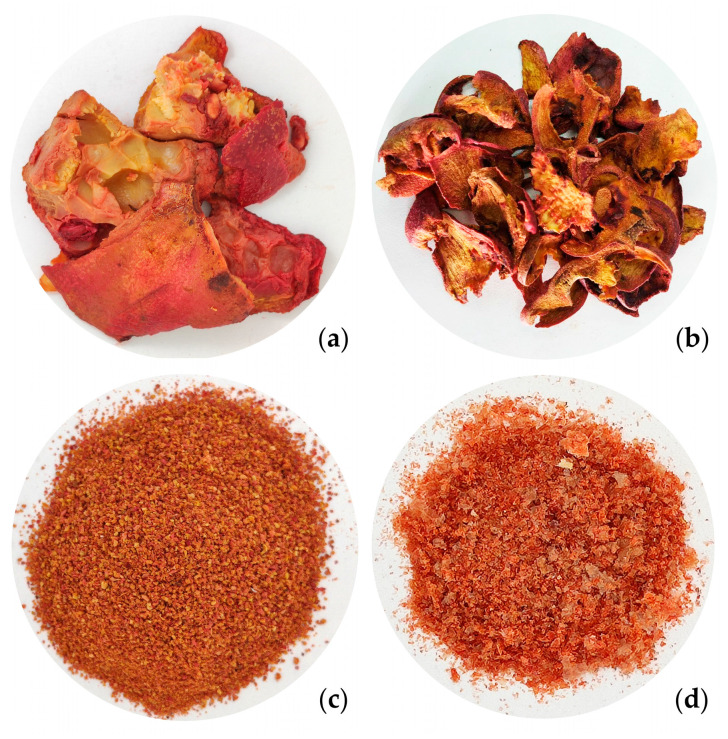
(**a**) Fresh pomegranate waste; (**b**) Dried pomegranate peel; (**c**) Pomegranate peel powder; (**d**) Dried pectin obtained at optimal extraction conditions.

Fresh pomegranate (*Punica granatum* var Wonderful) residue was obtained from a local company, Olivares de España S.R.L., located in Sarmiento, San Juan province, Argentina. The company produces pomegranates for sale as fresh and juice concentrate. The fresh peel (Figure 4a) was stored at 4 °C until their pretreatment. The treatment consists of manually removing the seeds and carpel membranes from the bio-waste to separate the peel. After that, the peel was dried in a solar dehydrator until the moisture content was less than 10% (Figure 4b), according to Capossio et al. [58]. Finally, the dehydrated peels were milled in a stainless steel blade grinder (TecnoDalvo, Model TDMC) and then sieved (25 mesh sieve) to obtain a uniform PPP (Figure 4c). For storage, the samples were placed in hermetically sealed plastic bags and kept in the dark at room temperature for one month.

### 3.2. Bio-Waste Physicochemical Characterization

The moisture content of the fresh peel and PPP was determined with a Radwag PMR50 moisture analyzer according to (AOAC Method 925.10) [59]. The ash content was analyzed following the AOAC Method 923.03 [59]. Protein content was determined via the Kjeldahl Method (AOAC Method 960.52) [59] and lipids via Soxhlet Extraction (AOAC Method 920.39) [59]. Lignin (standard method ASTM D1106) [60], cellulose (standard method ASTM D1103) [61], and holocellulose (standard method ASTM D1104) [62] contents were also determined, and hemicellulose content was calculated by the difference between holocellulose and cellulose. In addition, some physicochemical parameters of the residue were determined: pH of aqueous PPP solution at 1/20 ratio (AOAC Method 10.042) [59], water activity (a_w_) (VTSYIQI hygrometer of precision ± 0.02), titratable acidity (AOAC Method 942.15) [59], and soluble solids content (AOAC Method 932.12) [59]. All assays were performed in triplicate.

### 3.3. Pectin Extraction: Experimental Design

The pectin extraction from PPP was performed in triplicate according to the method proposed by Riveros-Gomez et al. [26]. PPP samples (0.5 ± 0.01 g) were placed and mixed in 15 mL Falcon tubes with citric acid solutions (pH 2.0, 2.5, and 3.0) at a fixed mass/volume ratio of 1/25 g/mL. Subsequently, they were immersed in an ultrasound bath preheated at different temperatures (70, 80, and 90 °C) and sonicated for 30, 45, and 60 min. After that, samples were centrifuged at 3000 rpm for 10 min to aid in the subsequent filtration process. The filtered liquid extract was collected in 15 mL Falcon tubes. To enhance the gelling of pectin, 5 mL of 96% ethanol was added to the extract, which was then refrigerated at 4 °C for 24 h. Subsequently, the obtained pectin was filtered using pre-weighed quantitative filters and washed with 70% ethanol to eliminate impurities. Finally, the pectin was dried at 50 °C. Then, the pectin extraction yield (η) was calculated according to Equation (3):

η [%] = (Dried pectin mass/Dried sample mass) × 100(3)

To carry out the experiments, a BBD has been used to identify the influence of three independent factors (concentration of the citric acid solution, temperature, and time) on the dependent variables: η and the TPCR (Table 1). BBD was chosen for its efficiency in terms of the relationship between the number of coefficients in the estimated model and the number of experiments conducted [63]. Experimental data were fitted following a multiple linear regression and then adjusted to an expression with the second-order polynomial expression (Equation (4)):

ŷ = b_0_ + Σ b_i_ x_i_ + Σ b_ij_ x_i_ x_j_ + Σ b_jj_ x_j_^2^ (4)

where b_0_, b_i_, b_ii_, and b_ij_ are regression coefficients for intercept, linear, quadratic, and interaction terms, respectively. x_i_ and x_j_ are values of the independent variables, while k equals the number of the tested factors (k = 3). 

The results of the objective functions (η and TPCR) were analyzed using the software (MATLAB Online https://la.mathworks.com/products/matlab-online/matlab-online-versions.html). Full quadratic models were fitted to the experimental responses using DOE (design of experiments)/ response surface analysis. To identify the significance of the effects and the interactions between them, an analysis of variance (ANOVA) was performed. The means were compared with Fisher’s test (*p* < 0.05), using the software InfoStat (https://www.infostat.com.ar/index.php?mod=page&id=37) 2017 [64]. 

### 3.4. Determination of Total Polyphenol Content (TPC)

The TPCR was calculated as the difference between the total polyphenol content (TPC) in the initial liquid extract (obtained by sonicating the PPP and citric acid during the time and at the temperature of each experiment before pectin precipitation) and the liquid phase remaining after precipitation and filtration of the pectin. The TPC was determined according to the Folin–Ciocalteu method [65].

### 3.5. Multi-Objective Optimization

One way to characterize the multi-objective optimization problem is by establishing a methodology to ascertain the values of specific decision variables that meet a specified condition. The phenolic-enriched pectin extraction process underwent multi-objective optimization using the genetic algorithm with Matlab Online software to find the optimal operational parameters. The objective functions are ${\hat{y}}_{\eta }$ and ${\hat{y}}_{TPCR}$, whose expression to be optimized is represented by Equation (5):

max (ŷ_η_, ŷ_TPCR_) = f (pH, T, t) (5)

Maximizing ŷ_η_ is considered one of the objective functions and undergoes minimization through the optimization procedure, thereby achieving the maximization of ${\hat{y}}_{\eta }$. Similarly, the ŷ_TPCR_ is subjected to optimization for maximization. During each iteration, the optimization parameters, including the pH of the extraction solution (pH), extraction time (t), and temperature (T), change, followed by the evaluation of the corresponding objective functions based on the updated values. The population size was 200, with the Pareto fraction set at 0.8 and 0.35, respectively. The optimization procedure concluded after 124 generations, when the average change in the dispersion of the Pareto solutions fell below 10-6 (termination tolerance).

### 3.6. Pectin Characterization

The pectin extracted at the optimal condition was dried at 50 °C for 24 h and saved (Figure 4d) for its characterization. The properties measured were ash content, equivalent weight (EW), methoxyl content (%Me), and degree of esterification (DE), according to Tripathi et al. [44], Nguyen and Pirak [46], and Yang et al. [66] Each determination was performed in triplicate (*n* = 3).

### 3.7. Identification and Quantification of Phenolics by HPLC-PDA-ESI-QTOF MS

Phenolic compounds contained in the initial liquid extract, and also in the liquid phase remaining after precipitation and filtration of the pectin, were obtained at optimal conditions. These analyses were performed by HPLC-PDA-ESI–MS/MS, using an Agilent Series 1200 LC System (Agilent, Santa Clara, CA, USA), coupled to a PDA detector (Agilent Series 1200) in tandem with an ESI source, connected to a MicroQTOF II (Bruker Daltonics, Billerica, MA, USA) mass spectrometer (MS and MS/MS). The HPLC system was equipped with a binary gradient pump, solvent degasser, and autosampler (Agilent Series 1200L, Santa Clara, CA, USA), as described by Fabani et al. [67].

## 4. Conclusions

As a result of the pomegranate processing industry (*Punica granatum* L., cv. Wonderful), the pomegranate peel is a by-product with potential use for the extraction of bioactive compounds and pectin. In the present work, pomegranate peel valorization was proposed to obtain polyphenols-enriched pectin by applying an alternative and more ecological extraction method that combines the use of citric acid as a hydrolytic agent and ultrasound. For this, pectin extraction was carried out under different times, temperatures, and pH conditions to determine the mathematical models, considering as response variables the extraction yield and the total phenolic content retained in pectin. A multi-objective optimization was applied using the response surface method to maximize the η and the TPCR functions.

After performing the optimization, multiple solutions were found, and the response variables for the solution selected as optimal were η = 31.89% and TPCR = 15.84 mg GAE/g pectin. At this point, the independent variables take the following values: time = 34.16 min, pH = 2.2, and temperature = 89.87 °C. According to the analysis of pectin characterization, it can be concluded that the pectin obtained is of low methoxyl and exhibits a medium value of the degree of esterification, with adequate pectin for forming gels in the presence of calcium or other divalent cations in a wide pH range, with or without sugar, and being suitable for low-sugar-content products and diet food production. 

It can be concluded that the studied process allows for the valorization of the pomegranate peel to obtain pectin enriched in polyphenols, an additive with high added and biological value with a potential use in the food industry, by using an eco-friendly method that reduces the environmental impact and the volume of waste, in line with the concept of circular economy and zero waste.

## Figures and Tables

**Table 1:** Fresh pomegranate physicochemical characterization.

Parameter	Value ^♠^
Moisture content [%]	60.62 ± 1.07
pH [dimensionless]	4.19 ± 0.03
Titratable acidity [%]	3.28 ± 0.12
Soluble solids [°Brix]	20.00 ± 0.01

^♠^ Average ± standard deviation (SD).

**Table 2:** Physicochemical composition of pomegranate peel powder on a dry basis.

Proximal Composition	Value ^♠^
Moisture [%]	6.67 ± 1.53
pH [dimensionless]	3.27 ± 0.12
a_w_ [dimensionless]	0.50 ± 0.02
Titratable acidity [%]	6.25 ± 0.44
Protein [%]	2.42 ± 0.07
Crude fiber [%]	8.16 ± 0.22
Ash [%]	2.69 ± 0.49
Lignin [%]	14.97 ± 1.03
Cellulose [%]	16.23 ± 0.19
Hemicellulose [%]	29.82 ± 0.30

^♠^ Average ± standard deviation (SD).

**Table 3:** Pectin quality parameters.

Parameter	Value ^1^
a_w_ [dimensionless]	0.51 ± 0.02
ash content [%]	2.55 ± 0.11
equivalent weight [g/mol]	175.27 ± 3.95
methoxyl content [%]	6.79 ± 0.08
Degree of esterification [%]	41.60 ± 0.49

^1^ Average ± standard deviation (SD).

## Data Availability

Data generated and analyzed during this study are included in this manuscript.

## Appendix A

**Table A1:** Experimental and adjusted η and TPCR.

Number	Independent Variables			Response Variables					
	t [min]	T [°C]	pH	Pectin Yield [%]			TPCR [mg GAE/g pectin]		
				Experimental	Adjusted	Residue	Experimental	Adjusted	Residue
1	30	80	2.0	24.17 ± 1.05	28.03	3.86	93.82 ± 1.05	80.64	13.18
2	30	80	3.0	12.12 ± 0.19	8.18	3.94	65.49 ± 0.53	57.52	7.97
3	60	80	2.0	37.55 ± 1.49	39.97	2.42	44.19 ± 0.95	51.24	7.05
4	60	80	3.0	15.13 ± 0.17	9.74	5.39	74.95 ± 0.17	87.22	12.27
5	45	70	2.0	42.47 ± 3.02	38.05	4.42	54.73 ± 0.45	67.40	12.67
6	45	70	3.0	8.12 ± 0.68	11.50	3.38	11.92 ± 1.04	18.92	6.99
7	45	90	2.0	47.03 ± 1.38	42.11	4.92	13.48 ± 2.40	5.57	7.91
8	45	90	3.0	15.69 ± 2.16	18.58	2.89	80.49 ± 1.54	66.91	13.58
9	30	70	2.5	18.34 ± 1.46	16.55	1.79	70.89 ± 1.48	69.93	0.96
10	60	70	2.5	24.06 ± 2.64	23.89	0.17	53.89 ± 2.72	33.18	20.71
11	30	90	2.5	24.09 ± 1.76	22.72	1.37	6.31 ± 0.22	26.12	19.81
12	60	90	2.5	28.86 ± 2.61	28.88	0.02	63.10 ± 2.42	63.17	0.07
13	45	80	2.5	19.66 ± 2.28	18.89	0.76	50.56 ± 5.20	50.10	0.45

**Table A2:** ANOVA of η and TPCR of regression models.

Response Variables	Variation Source	Sum of Squares	Degrees of Freedom	Mean Square	Calculated *F* Value	Tabulated *F* Value, *F* (9,29, 0.05)
Pectin yield	regression	4635.88	9	515.10	32.18	2.223“significant”
	residues	464.22	29	16.01		
	total	5100.10	38			
	R^2^	0.909				
	adjusted R^2^	0.881				
TPCR	regression	21,848.22	9	2427.58	13.42	2.223“significant”
	residues	5247.62	29	180.95		
	total	27,095.84	38			
	R^2^	0.806				
	adjusted R^2^	0.746				

**Table A3:** Statical parameters of each of the terms and their effect on the stored significance for η and TPCR models.

Term	η [%]				TPCR [mg GAE/g pectin]			
	Coefficient	T-Value	*p*-Value	VIF	Coefficient	T-Value	*p*-Value	VIF
Constant	440.000	8.51	0.000		1074.000		0.000	
pH	−92.900	−15.33	0.000	1.00	−628.000	1.21	0.235	1.00
t	1.640	4.15	0.000	1.00	−20.250	−0.02	0.987	1.00
T	−8.170	3.51	0.001	1.00	5.560	−1.28	0.212	1.00
pH pH	14.270	2.33	0.027	1.35	21.300	1.04	0.308	1.35
t t	−0.004	−0.64	0.526	1.35	0.061	2.67	0.012	1.35
T T	0.051	3.34	0.002	1.35	−0.157	−3.06	0.005	1.35
pH t	−0.346	−2.24	0.033	1.00	1.970	3.80	0.001	1.00
pH T	0.151	0.65	0.519	1.00	5.491	7.07	0.000	1.00
t T	−0.002	−0.26	0.799	1.00	0.123	4.75	0.000	1.00

Complete second-order polynomials of pectin yield (Equation (A1)) and TPCR (Equation (A2)) models:

Pectin yield [%] = 440.000 − 92.900 pH + 1.640 t + 8.171 T + 14.270 pH^2^ − 0.004 t^2^ + 0.051 T^2^ − 0.346 pH t + 0.151 pH T − 0.002 t T (A1)

TPCR [mg GAE/g pectin] = 1074.000 − 628.000 pH − 20.250 t + 5.560 T + 21.300 pH^2^ + 0.061 t^2^ − 0.016 T^2^ + 1.970 pH t + 5.491 pH T + 0.123 t T (A2)
